# Changes in aortic growth rate and factors influencing aneurysmal dilatation after uncomplicated acute type B aortic dissection

**DOI:** 10.1093/icvts/ivac126

**Published:** 2022-05-05

**Authors:** Jae Hang Lee, Joon Chul Jung, Bongyeon Sohn, Hyoung Woo Chang, Dong Jung Kim, Jun Sung Kim, Cheong Lim, Kay-Hyun Park

**Affiliations:** Department of Thoracic and Cardiovascular Surgery, Seoul National University Bundang Hospital, Seoul National University College of Medicine, Seongnam, South Korea

**Keywords:** Uncomplicated type B aortic dissection, Aortic growth rate, Aneurysmal change, Thoracic endovascular aortic repair

## Abstract

**OBJECTIVES:**

The aim of this study was to evaluate changes in aortic growth rate and factors influencing aneurysmal dilatation after uncomplicated acute type B aortic dissection (ABAD).

**METHODS:**

Medically treated patients with uncomplicated ABAD between September 2004 and January 2020 were retrospectively reviewed. Diameters of 6 different sites in the descending aorta were measured and aortic growth rate was calculated according to the time interval. Factors associated with aneurysmal changes were also investigated.

**RESULTS:**

This study enrolled a total of 105 patients who underwent >2 serial computed tomography with a mean follow-up duration of 35.4 (12.1–77.4) months. The mean overall growth rates of the proximal descending thoracic aorta (DTA), mid-DTA, distal DTA, proximal abdominal aorta, maximal DTA and maximal abdominal aorta were 0.6 (1.9), 2.9 (5.2), 2.1 (4.0), 1.2 (2.2), 3.3 (5.6) and 1.4 (2.5) mm/year, respectively. The growth rate was higher at the early stage. It decreased over time. Growth rates of proximal DTA, mid-DTA, distal DTA, proximal abdominal aorta, maximal DTA, and maximal abdominal aorta within 3 months after dissection were 1.3 (9.6), 12.6 (18.2), 7.6 (11.7), 5.9 (7.5), 16.7 (19.8) and 6.8 (8.9) mm/year, respectively. More than 2 years later, they were 0.2 (0.6), 1.6 (1.6), 1.2 (1.3), 0.9 (1.4), 1.7 (1.9) and 1.2 (1.7) mm/year, respectively. Factors associated with aneurysmal changes after uncomplicated ABAD included an elliptical true lumen (odds ratio = 3.16; 95% confidence interval: 1.19–8.41; *P *=* *0.021) and a proximal entry >10 mm (odds ratio = 3.08; 95% confidence interval: 1.09–8.69; *P *=* *0.034) on initial computed tomography imaging.

**CONCLUSIONS:**

The aortic growth rate was higher immediately after uncomplicated ABAD but declined eventually. Patients with an elliptical true lumen and a large proximal entry might be good candidates for early endovascular intervention after uncomplicated ABAD.

**Figure:**
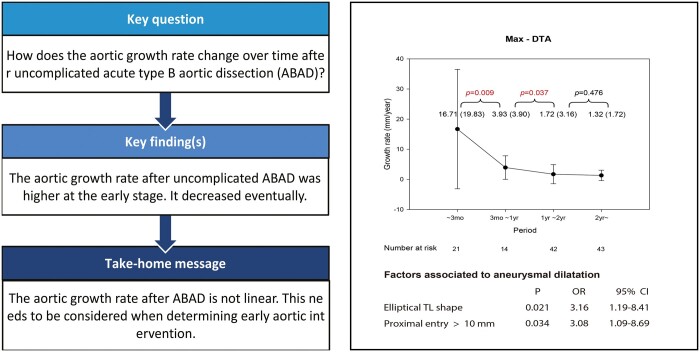

## INTRODUCTION

Acute type B aortic dissection (ABAD) is a catastrophic cardiovascular condition associated with high morbidity and mortality. However, ABAD is associated with better survival than acute type A aortic dissection. Patients with uncomplicated ABAD are mainly treated conservatively [1–4]. However, aortic aneurysmal changes are found in 20–80% of patients during follow-up and the risk of rupture increases accordingly [5–10]. Some studies have recently highlighted the importance of early intervention even in uncomplicated ABAD for this reason [11]. Thoracic endovascular aortic repair (TEVAR) is strongly recommended for complications such as malperfusion, refractory pain, uncontrolled hypertension and impending rupture associated with ABAD [12–17].

Recently, guidelines and studies have shown the benefit of TEVAR in high-risk patients of uncomplicated ABAD [11, 18]. Even in low-risk patients with uncomplicated ABAD, early aortic intervention can facilitate positive aortic remodelling. Therefore, many interventionists rush to endovascular procedure after confirming that aortic growth is progressing after a short-term follow-up [19]. However, the change in aortic growth rate after ABAD is patient dependent, and uncomplicated ABAD yields good results following medical treatment alone. Thus, it is crucial to select appropriate patients for early aortic endovascular procedures.

Therefore, the objective of this study was to evaluate changes in aortic growth rate after uncomplicated ABAD and to identify factors associated with favourable results following early aortic intervention.

## MATERIALS AND METHODS

### Ethics statement

Our institutional review committee approved this retrospective study (IRB approval number: B-2101-661-104). The need for informed consent was waived because of the retrospective nature of this study. We present this article in accordance with the Strengthening the Reporting of Observational Studies in Epidemiology guideline checklist.

### Population

**Figure 1:**
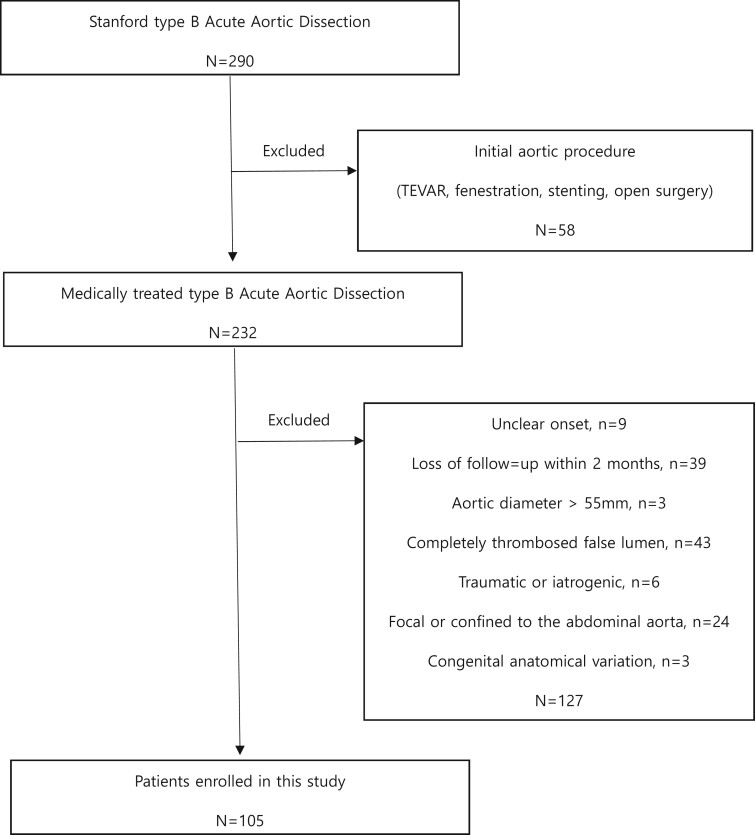
Enrolment of patients.

Patients with uncomplicated ABAD who underwent >2 serial computed tomography (CT) imaging were studied from September 2004 to January 2020. Uncomplicated ABAD was defined as the absence of organ malperfusion and presenting within 14 days of symptom onset. Patients who underwent endovascular treatment such as TEVAR and fenestration during their initial hospitalization were excluded. Other exclusion criteria were: (i) an unclear onset, (ii) an initial aortic diameter of >55 mm, (iii) a completely thrombosed false lumen (FL) or intramural haematoma, (iv) traumatic or iatrogenic dissection, (v) ABAD confined to the abdominal aorta or (vi) ABAD combined with a congenital anatomical variation such as an aberrant subclavian artery or coarctation of aorta (Fig. 1).

### Review of computed tomography images

**Figure 2:**
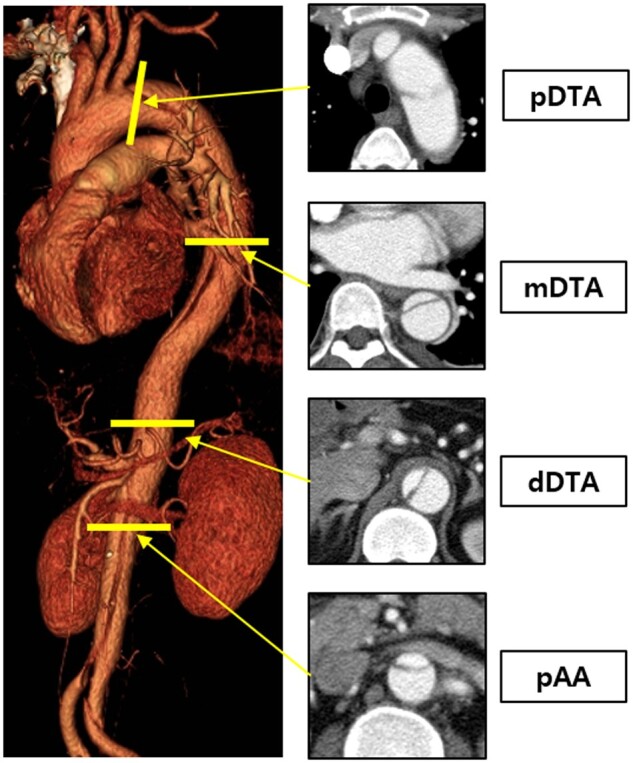
Measurement of aortic diameter at different levels.

Two researchers (Jae Hang Lee and Bongyeon Sohn) reviewed CT scans using INFINITT PACS (INFINITT Healthcare Co. Ltd., Seoul, South Korea). We reviewed CT images performed on the day of admission for aortic dissection in all patients and measured the tear site, true lumen (TL) and FL conditions, and the size of the proximal entry. The aortic diameter was measured by CT at the time of admission to the emergency room, within 3 months, within 1 year, within 2 years and the most recent CT. The growth rate between each period of CT was calculated. The location of the tear site and the size of proximal entry were measured by evaluating axial, coronal, and sagittal images. If elliptical configuration of the TL was observed at any level in descending thoracic aorta (DTA), it was defined as elliptical TL. The diameter was measured at a total of 6 levels. The proximal DTA (pDTA) was measured immediately below the left subclavian artery (LSCA). The mid-DTA (mDTA) was measured at the left lower pulmonary vein level. The distal DTA (dDTA) was measured above the coeliac artery. The proximal abdominal aorta (pAA) was measured below the lowest renal artery (Fig. 2). Regardless of the level, the diameter with the largest area in the thoracic aorta [maximal DTA (maxDTA)] and the diameter with the largest area in the maximal abdominal aorta (maxAA) were measured. The diameter was measured in the axial view perpendicular to the direction of the aorta. The diameter was measured including the outer wall. The direction of the diameter measured at each level was established similar to serial follow-up CT images.

### Statistical analysis

Categorical variables are expressed as numbers (%). Continuous variables are reported as mean (standard deviation) or median (interquartile range) and compared by Student's *t*-test or Mann–Whitney *U*-test. SPSS 19.0 for Windows (SPSS Institute, Chicago, IL, USA) was used for all statistical analyses. Shapiro–Wilk test was performed to evaluate the normality of continuous variables. According to the test for normality distribution, either Student’s *t*-test or Mann–Whitney *U*-test was used for the comparison of growth rates at different time intervals. Univariable and multivariable logistic regression analyses for rapid (>5 mm/year) aortic growth rate in 2–3 years were performed. Variables that showed a *P*-value of <0.2 in the univariable analyses were chosen for multivariable analysis. In the multivariable analysis, backward conditional variable selection method was applied with the probability for the entry of 0.05 and the removal of 0.10.

## RESULTS

### Baseline characteristics and radiological findings

A total of 105 medically treated patients were enrolled in this study. All patients were Asians, including 82 (78.1%) male patients. The median age of all patients was 51 (44–63) years. The most common comorbidity was hypertension (*n* = 77, 73.3%). Based on initial radiographic findings, 41 (39.0%) patients had a maximal aortic diameter >40 mm. Intimal tears were observed at pDTA in 67 (63.8%) patients. An elliptical TL and a patent FL were observed in 37 (35.2%) and 55 (52.4%) patients, respectively. Twenty-four (22.9%) patients had a proximal tear of larger than 10 mm. The mean follow-up duration was 35.4 (12.1–77.4) months. Additional baseline characteristics are presented in Table 1.

**Table 1:** Patient characteristics

Variables	Number of patients (*n* = 105)
Ethnicity, Asian, *n* (%)	105 (100)
Male sex, *n* (%)	82 (78)
Age (years), median (IQR)	51 (44–63)
Hypertension, *n* (%)	77 (73)
Diabetes, *n* (%)	12 (11)
Dyslipidaemia, *n* (%)	19 (18)
Coronary artery disease, *n* (%)	5 (5)
Chronic renal failure, *n* (%)	4 (4)
Marfan syndrome, *n* (%)	4 (4)
Radiologic findings	
Initial diameter >40 mm, *n* (%)	41 (39)
Proximal DTA tear, *n* (%)	67 (64)
Elliptical TL shape, *n* (%)	37 (35)
Absence of thrombus in FL, *n* (%)	55 (52)
Proximal entry >10 mm, *n* (%)	24 (23)

DTA: descending thoracic aorta; FL: false lumen; IQR: interquartile range; TL: true lumen.

### Changes in aortic growth rates

**Figure 3:**
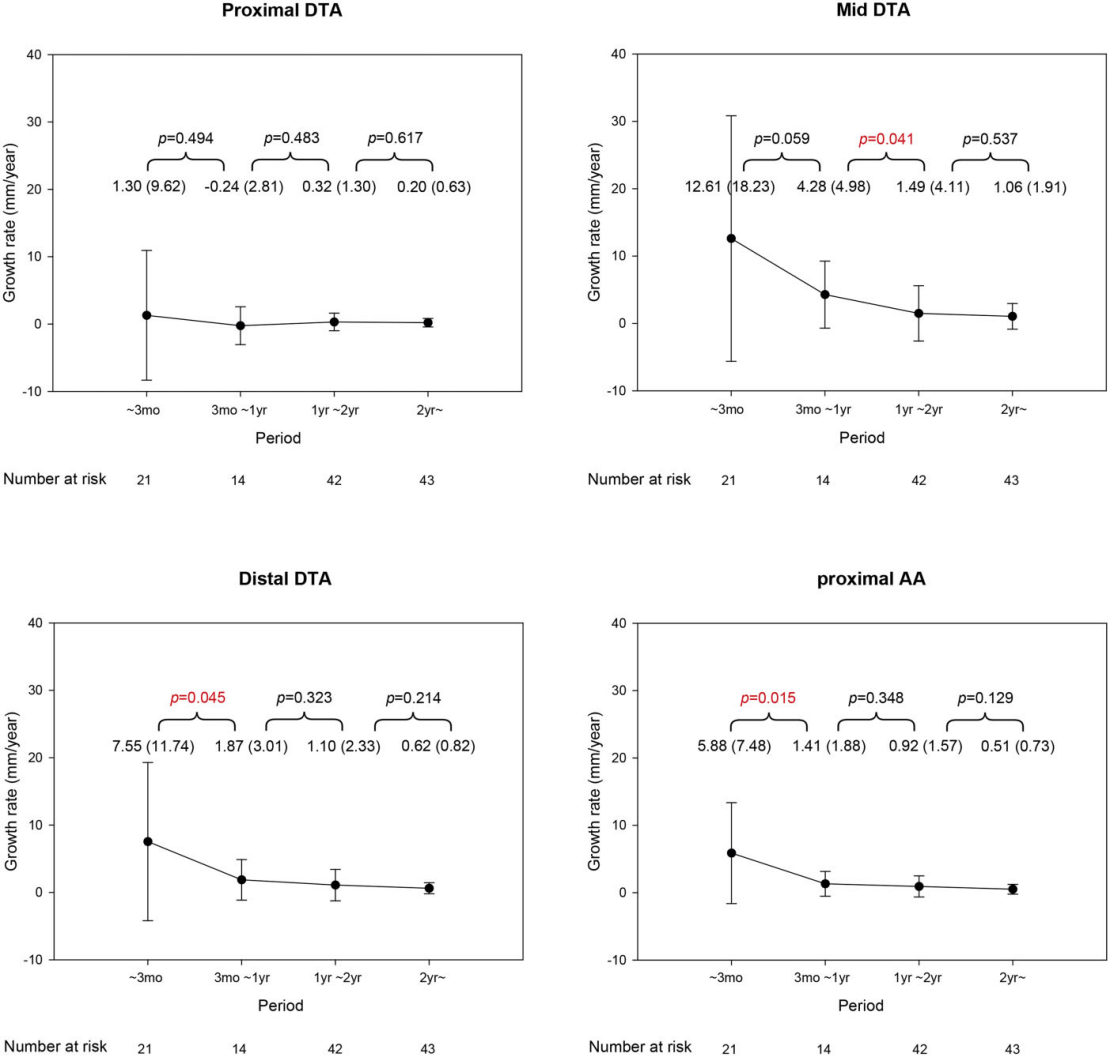
Temporal variation in aortic growth rate at different levels.

Overall growth rates of pDTA, mDTA, dDTA, pAA, maxDTA and maxAA were 0.6 (1.9), 2.9 (5.2), 2.1 (4.0), 1.2 (2.2), 3.3 (5.6) and 1.4 (2.5) mm/year, respectively. The growth rate of pDTA was low. Thus, no difference in growth rate was found during the follow-up period. In contrast, the mDTA increased rapidly to 12.6 (18.2) mm/year in the first 3 months. It then decreased to 4.3 (5.0) mm/year in the first-year follow-up. The rate gradually declined to 1.5 (4.1) mm/year at the second year, which showed a statistically significant difference compared to the first-year rate (*P *=* *0.041). The dDTA increased rapidly to 7.6 (11.7) mm/year in the initial 3 months. It then decreased to 1.9 (3.0) mm/year at 1 year, which was statistically significant compared to the rate at 3 months (*P *=* *0.045). At the second year, the aortic growth rate was further reduced to 1.1 (2.3) mm/year. The pAA rapidly increased to 5.9 (7.5) mm/year at the first 3 months. It then declined to 1.4 (1.9) mm/year at the first year, which was statistically significant compared to the rate at 3 months (*P *=* *0.015). At the second year, the aortic growth rate declined further to 0.9 (1.6) mm/year (Fig. 3).

**Figure 4:**
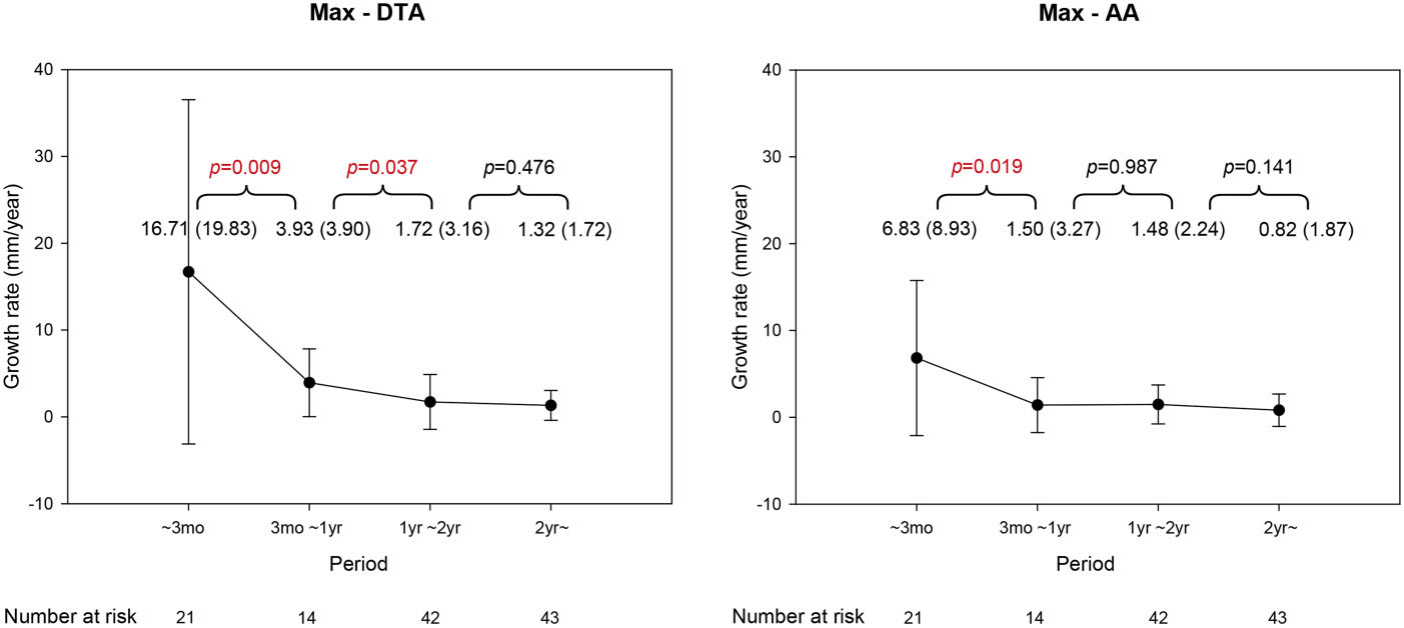
Temporal variation in the maximal thoracic and abdominal aortic growth rates.

The maxDTA rapidly increased to 16.7 (19.8) mm/year at 3 months. It then decreased to 3.9 (3.9) mm/year at the first year. It declined eventually to 1.7 (3.2) mm/year at the second year. Both the first interval (*P *=* *0.009) and the second interval (*P *=* *0.037) showed statistically significant differences. The maxAA rapidly increased to 6.8 (8.9) mm/year at 3 months and then decreased to 1.5 (3.3) mm/year at 1 year, which was statistically significant compared to the rate at 3 months (*P *=* *0.019). The aortic growth rate declined further to 1.5 (2.2) mm/year at the second year (Fig. 4).

### Factors associated with aneurysmal dilatation

Aortic growth rate of >5 mm/year during the first 2–3 years was considered to be an aneurysmal dilatation. According to results of univariable analyses, elliptical TL shape, absence of thrombus in FL and proximal entry tear size >10 mm showed a *P*-value of <0.2. Thus, they were analysed in the multivariable analysis. In the final model, 2 variables (elliptical TL shape and proximal entry >10 mm) were included. Both elliptical TL shape (odds ratio = 3.16; 95% confidence interval: 1.19–8.41; *P *=* *0.021) and proximal entry >10 mm (odds ratio = 3.08; 95% confidence interval: 1.09–8.69; *P *=* *0.034) were factors associated with aneurysmal dilatation after uncomplicated ABAD (Table 2).

**Table 2:** Factors associated with aneurysmal dilatation

	Univariable analysis			Multivariable analysis		
	*P*-Value	OR	95% CI	*P*-Value	OR	95% CI
Initial diameter >40 mm	0.295	1.62	0.66–4.02			
Proximal DTA tear	0.331	1.63	0.61–4.34			
Elliptical TL shape	0.004	3.96	1.55–10.11	0.021	3.16	1.19–8.41
Absence of thrombus in FL	0.022	2.94	1.17–7.42			
Proximal entry >10 mm	0.006	4.05	1.51–10.88	0.034	3.08	1.09–8.69

CI: confidence interval; DTA: descending thoracic aorta; FL: false lumen; OR: odds ratio; TL: true lumen.

## DISCUSSION

Key points of this study could be summarized as follows: (i) the aortic growth rate after uncomplicated ABAD was not linear (the aortic growth rate after uncomplicated ABAD was increased at the early stage, but declined eventually) and (ii) predictors of aneurysmal change after ABAD included an elliptical TL and a large proximal entry on initial CT imaging. These results need to be considered when determining early aortic intervention after uncomplicated ABAD.

Uncomplicated ABAD is an aortic disease with low in-hospital mortality. However, it can lead to various changes in the downwards aorta during the follow-up period [1–4]. Although aortic growth or aneurysmal change can be found in many cases, its rate is patient dependent. In contrast, a complete positive remodelling of the downward aorta might be observed occasionally. Various studies have demonstrated that characteristics of patients and the anatomy of the aorta determine the growth rate [7–9]. Some studies have investigated the aortic growth rate after ABAD. However, most of previous studies that reported aortic growth rates were only based on linear growth patterns by comparing initial and final CTs [9].

Studies reporting temporal variation in aortic growth rate are very rare. Hosn *et al.* [20] have found that the growth rate of DTA is different between the first and second intervals. However, the timing of the first and second interval CTs was not standardized. In addition, only 2 intervals were compared in their study. In contrast, our study standardized follow-up intervals at 3 months, 1 year, 2 years and >2 years. Peterss *et al.* [21] have presented similar results to our study. They described differences in aortic growth rates according to acute/subacute/chronic phases and showed that the aortic growth rates in the acute phase were very rapid. Their study differed from ours in that they set time points to be 2 weeks and 3 months after the onset of ABAD, whereas we measured diameters at different points.

In this study, we demonstrated that the aortic growth during the follow-up period of uncomplicated ABAD did not show a linear pattern (i.e. the initial aortic growth rate was very high, it then decreased with time). Although histological changes after acute aortic dissection have been studied, detailed patterns that change over time have not yet been reported. However, many researchers have explained that various pathological changes can affect the overall integrity of the aorta [21]. The pattern of decreasing aortic growth rate would be attributed to the rapid increase in the aortic diameter due to the weakened aortic wall, especially for an unstable FL at the initial stage of aortic dissection and the slow growth rate as the adventitia becomes solid and stabilizes over time.

TEVAR for ABAD is showing good results. In particular, the role of TEVAR for complicated ABAD or ABAD with aneurysmal change on initial CT is clear [22]. However, according to our study, we think there is controversy in performing TEVAR for uncomplicated ABAD with small aortic diameter only because of the rapid increase in aortic diameter in the short-term follow-up (even for patients exceeding 0.5 cm at 6 months), and further investigation is needed. Similar to our argument, a recent study has revealed that after conversion to TEVAR in conservatively pretreated chronic type B dissections, a more pronounced decrease in the diameter of the descending aorta than in patients treated in the acute phase is observed [23].

Notably, our study showed fewer changes in the diameter of pDTA because the diameter was measured immediately below the LSCA, which is more proximal than the intimal tear. Thus, it is difficult to implicate it in the dissection. If the pDTA level was measured from the straight portion over the aortic curvature, the pDTA might show a higher growth rate than the mDTA. This finding can also be interpreted in other ways. When performing TEVAR in ABAD patients, the LSCA level used as the proximal landing zone changes little in diameter with the passage of time. Thus, the aortic diameter of the LSCA level represents a robust parameter for stent graft sizing.

Results of our study suggest that factors associated with aneurysmal dilatation in the radiologic findings include an elliptical TL and a proximal large entry, which are frequently reported in many previous studies. An elliptical TL indicates increased pressurization of the FL, which induces higher radial force in the FL and causes aneurysmal dilatation [24, 25]. A proximal large entry can also increase the pressure of the FL, which similarly increases the aortic growth rate [26]. However, patent FL generally suggested as a risk factor in other existing studies [27] did not appear to be an important risk factor in our study probably because patients with complete thrombosis of the FL were excluded from this study.

### Limitations

This study has several limitations. It was a single-centre, retrospective observational study that enrolled a relatively small number of Asian patients. In addition, most CT scans were non-ECG-gated, making it difficult to accurately measure the aortic diameter in the axial view of the CT. We calculated the aortic diameter including the outer wall as the largest short-axial diameter. However, errors due to aortic tortuosity were not eliminated. We measured the diameter in only the axial view because patients arriving from local clinics underwent CTs performed via different modalities. In addition, it was technically difficult to perform measurements based on the centreline due to characteristics of aortic dissection. Instead, while reviewing serial CT imaging of each patient, we minimized the error by measuring the aortic diameter at the same level and in the same direction as in the initial CT. In addition, the fact that only 2 researchers performed all image reviews could be a limitation. Statistically, a paired test is optimal for comparing variables that are correlated with each other. However, paired tests could not be conducted because of missing values. We calculated the growth rates between 5 time points and compared the mean growth rates back and forth using the Student’s *t*-test. Although this study tried to investigate the correlation between radiological characteristics and aneurysmal changes, since only the radiological variables were included and the patient clinical characteristics and history were excluded, bias may have been present in the risk factors identified for the aneurysmal dilatation. Finally, the evenness of the interval was arbitrary. We tried to find out that the aortic growth rate was very high immediately after uncomplicated ABAD by following up at the initial 3-month interval rather than the regular 1-year interval.

## CONCLUSIONS

In our experience, the aortic growth rate after uncomplicated ABAD is not linear. The aortic growth rate after uncomplicated ABAD was higher at the early stage. It then decreased over time. Factors associated with aneurysmal dilatation after ABAD included an elliptical TL and a large proximal entry on initial CT imaging. These results might need to be considered when determining early aortic intervention after uncomplicated ABAD. Further investigation is needed.

**Conflict of interest:** none declared.

## Data Availability Statement

The data underlying this article will be shared upon reasonable request to the corresponding author.

## Author contributions

**Jae Hang Lee:** Conceptualization; Data curation; Formal analysis; Investigation; Methodology; Project administration; Visualization; Writing – original draft. **Joon Chul Jung:** Data curation; Investigation. **Bongyeon Sohn:** Data curation; Investigation. **Hyoung Woo Chang:** Formal analysis; Investigation; Methodology; Visualization. **Dong Jung Kim:** Conceptualization; Supervision. **Jun Sung Kim:** Conceptualization; Supervision. **Cheong Lim:** Conceptualization; Supervision. **Kay-Hyun Park:** Conceptualization; Supervision.

## Reviewer information

Interactive CardioVascular and Thoracic Surgery thanks Luca Bertoglio, Sven Peterss, Gabriele Piffaretti, Thomas Schachner and the other, anonymous reviewer(s) for their contribution to the peer review process of this article.

## ABBREVIATIONS

ABAD: Acute type B aortic dissection
CT: Computed tomography
dDTA: Distal descending thoracic aorta
DTA: Descending thoracic aorta
FL: False lumen
LSCA: Left subclavian artery
maxAA: Maximal descending thoracic aorta
maxDTA: maximal abdominal aorta
mDTA: Mid-descending thoracic aorta
pAA: Proximal abdominal aorta
pDTA: Proximal descending thoracic aorta
TEVAR: Thoracic endovascular aortic repair
TL: True lumen
